# Effects of Endurance Exercise Modalities on Arterial Stiffness in Patients Suffering from Unipolar Depression: A Randomized Controlled Trial

**DOI:** 10.3389/fpsyt.2017.00311

**Published:** 2018-01-22

**Authors:** Henner Hanssen, Alice Minghetti, Oliver Faude, Arno Schmidt-Trucksäss, Lukas Zahner, Johannes Beck, Lars Donath

**Affiliations:** ^1^Department of Sport, Exercise and Health, University of Basel, Basel, Switzerland; ^2^Klinikum Sonnhalde, Psychiatrie und Psychotherapie, Riehen, Switzerland; ^3^Institute of Exercise Training and Computer Science in Sport, German Sport University Cologne, Köln, Germany

**Keywords:** depression, high-intensity exercise training, cardiovascular risk, arterial stiffness, prevention

## Abstract

**Background:**

Psychiatric disorders are associated with a higher prevalence of cardiovascular disease and mortality. Regular exercise has been shown to reduce depressive symptoms and improve arterial stiffness as a biomarker of cardiovascular risk. We aimed to investigate the effects of different exercise modalities on depression severity index and arterial stiffness in patients suffering from unipolar depression.

**Methods:**

34 patients suffering from unipolar depression [female: 25, male: 9, age: 37.8, Beck-Depression-Inventory-II (BDI-II) score: 31.0] were enrolled in this two-armed randomized controlled trial. Central hemodynamics, augmentation index at heart rate 75/min (AIx@75) and aortic pulse wave velocity (PWV) were obtained by an oscillometric monitoring device. Maximal bicycle ergometer exercise testing yielded maximal fitness parameters. Patients were assigned to either high-intensity low volume (HILV) or moderate continuous aerobic training (MCT). Both intervention groups trained three times a week during a 4-week intervention period. BDI-II were filled out by the patients before and after the intervention period.

**Results:**

We found moderate interaction effects on depression severity reduction $\left(\eta_{p}^{2}=0.10\right)$. HILV showed a 85% beneficial effect in lowering BDI-II scores compared to MCT (HILV: pre: 28.8 (9.5), post: 15.5 (8.5), SMD = 1.48), MCT: (pre: 33.8 (8.5), post: 22.6 (7.5), SMD = 1.40). Reduction of AIx@75 was more pronounced after MCT (SMD = 0.61) compared to HILV (SMD = 0.08), showing 37% possibly beneficial effects of MCT over HILV. PWV remained unchanged in both training groups.

**Conclusion:**

Both training regimes showed large effects on the reduction of depressive symptoms. While HILV was more effective in lowering depression severity, MCT was more effective in additionally lowering peripheral arterial stiffness. Exercise should be considered an important strategy for preventive as well as rehabilitative treatment in depression.

## Introduction

Depression is a widespread public-health problem and one of the leading causes of disease burden worldwide (1) affecting an estimated 350 million people (2). Depression affects health status more than somatic diseases such as coronary artery disease (CAD), diabetes, or musculoskeletal disease such as arthritis (1). The overall mortality rate of the disease lies around 4% (3). Depression is characterized by a distinct change of mood such as sadness or irritability and is accompanied by several psychophysiological alterations including disturbance in sleep, appetite or sexual desire, constipation, loss of ability to experience pleasure, crying, slowing of speech and action as well as suicidal thoughts (4). Guidelines from the National Institute of Health and Care Excellence (NICE) recommend cognitive behavioral therapy as treatment of choice for mild to moderate depression, followed by antidepressant medication (3). NICE and WHO guidelines both recommend regular exercise, in the standard treatment of depression (2, 3, 5).

Growing evidence from cross-sectional and longitudinal studies show that physically active people are at lower risk of developing depression (6). A recent meta-analysis compared the efficacy of exercise for patients suffering from unipolar depression to the most common alternative treatment strategies, such as psychological treatment, antidepressant medication, and usual care. The meta-analysis revealed that exercise has a moderate to large effect compared to control conditions and it yielded a moderate effect as an adjunct to antidepressant medication (5).

Depression can be diagnosed in patients with CAD (7). A number of prospective studies have found an association between depression and increased mortality in a variety of CAD populations (8). Depression is linked to traditional cardiovascular risk factors such as hypertension, diabetes, and insulin resistance (9) as well as alterations in immune response and inflammation (10). A population-based cross-sectional study found that patients with increased arterial stiffness were more likely to demonstrate depressive symptoms (11). Arterial stiffness is an established, independent predictor of cardiovascular events and stroke in healthy patients (12). The augmentation index (AIx) serves as peripheral hemodynamic parameter closely related to several risk factors of atherosclerosis and future cardiovascular events (13). Higher levels of physical activity and fitness have been linked to lower central arterial stiffness measured by AIx (14) as well as to a reduction in all-cause mortality and cardiovascular disease (CVD) mortality (15). Thus, aerobic endurance exercise, even at higher intensities, can be recommended as treatment strategy in CAD patients (15). Considering strategies to maximize benefits that not only reduce depressive symptoms but also improve biomarkers of increased cardiovascular risk are of high clinical relevance. Exercise therapy is gaining much interest as a treatment option for depression and is a recommended approach in CVD.

To date, most exercise intervention studies investigating the effects of exercise on depression have examined single exercise intensities and continuous application forms (16–18). Recent studies have demonstrated that interval exercise at high intensity (80–90% of VO_2max_) is superior to continuous exercise at a moderate intensity (50–60% VO_2max_) for increasing VO_2max_ in stable patients with CAD (15) as well as in healthy subjects (19). The relationship between continuous steady-state exercise and high-intensity intervals and their effect on unipolar depression have yet to be systematically evaluated. In this study, moderate continuous aerobic training (MCT) was regarded as control vs. low volume, high-intensity aerobic training applied in intervals [high-intensity low volume (HILV)].

## Materials and Methods

### General Study Design and Participants

**Figure 1:**
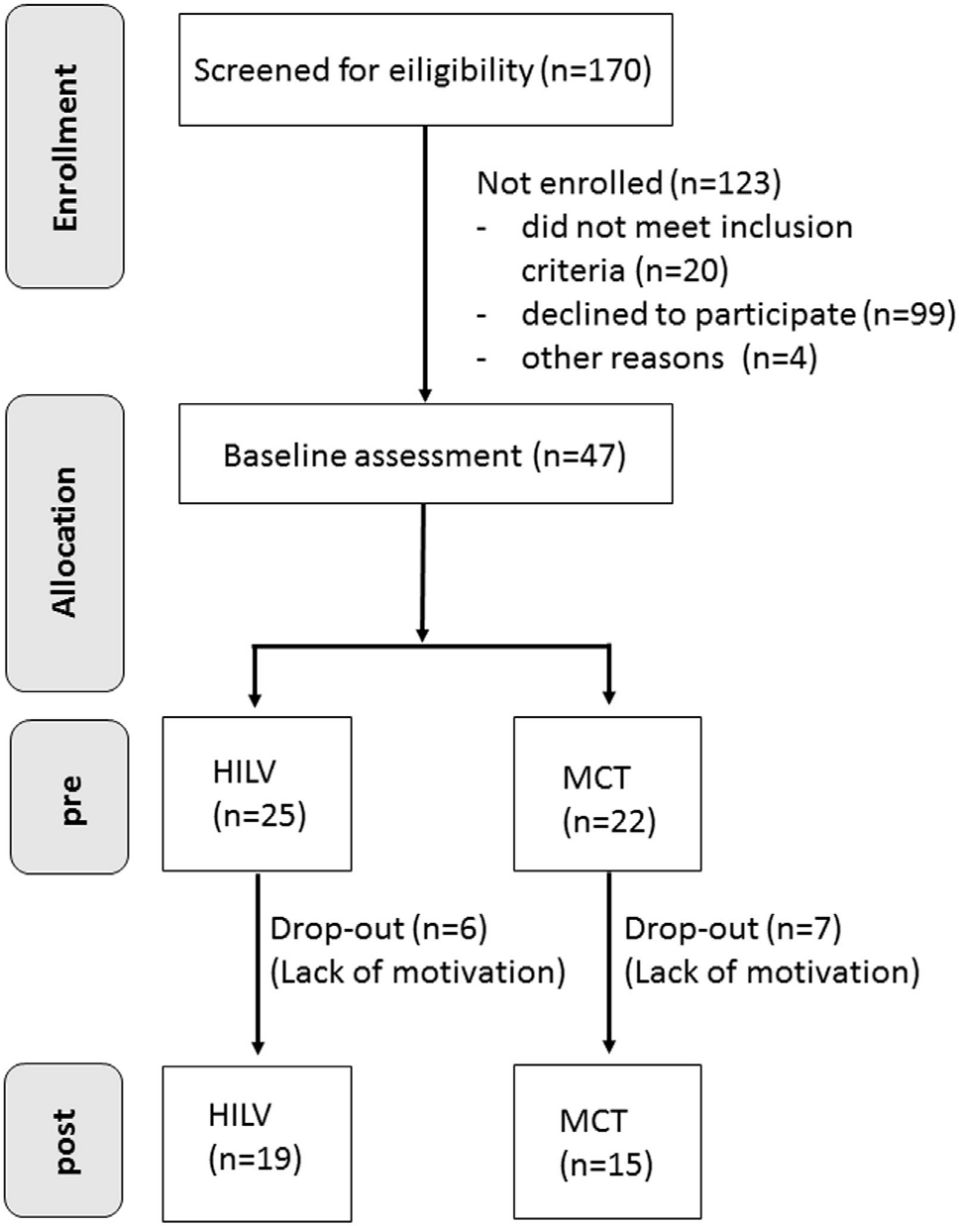
Flow Chart of the Randomized Controlled Trial.

The present study was designed as a two-armed randomized clinical trial. The participants were stationary patients at the Clinic Sonnenhalde in Riehen, Switzerland, where they received all study-related information and signed a written consent form before participating. After pre-testing procedures to determine the clinical baseline for disease severity, a total of 34 patients were included in the study. Due to the fact that patients suffered from unipolar depression and were hospitalized for this reason, a relatively large number of inpatients was not willing to undergo arterial stiffness measurements or exercise testing. Some patients preferred not to participate in all measurements, for example, if they were not feeling well on the day the measurements were scheduled. This explains why, out of 170 patients, 99 were not willing to participate in the study and were not enrolled (Figure 1). The patients were randomly assigned [minimization method (20), strata: age, gender, BMI, depression severity] either to the HILV group or the moderate, continuous aerobic training group (MCT) (see Table 1). Both groups exercised over a period of 4 weeks, three times a week. Post-testing occurred after the completion of a total of 12 training sessions. Pharmacological medication was issued to the patients according to the physicians’ recommendations. Inpatients had scheduled visits with psychologists and in groups. The intake of pharmaceutical medication was comparable in both groups (Table 2). Before and after the 4 weeks of supervised training, arterial stiffness assessment, cardiopulmonary exercise testing, and depression index assessments were conducted. Post intervention assessment was performed in an identical manner as the pre-measurement procedures.

**Table 1:** Baseline data of the participants for both intervention groups (HILV and MCT).

	HILV (*n* = 19)	MCT (*n* = 15)
Gender [m/f]	5/14	4/11
Age [years]	38.1 (12.2)	37.5 (10.1)
BMI [kg m^−2^]	22.6 (3.3)	24.9 (5.2)
Systolic BP [mmHg]	120.1 (10.5)	115.9 (11.2)
Diastolic BP [mmHg]	97.4 (8.7)	92.9 (10.0)
BDI-II [score]	28.8 (9.5)	33.8 (8.5)

*Data are provided as means with SDs*.

*Gender is indicated as f, female; m, male; BMI, body mass index; BP, blood pressure; BDI-II, Beck-Depression-Inventory second edition, expressed in BDI-II score; HILV, high-intensity low volume*.

**Table 2:** Medication use in high-intensity low volume (HILV) and MCT.

	HILV (*n* = 19)	MCT (*n* = 15)
SNRIs	4	5
Venlafaxin (75, 175, 225 mg)	2	4
Mirtzapin (7.5, 15, 30 mg)	2	0
Wellbutrin (150 mg)	0	1
SSRIs	10	13
Trittico (25, 50, 100 mg)	2	4
Escitalopram (10, 20 mg)	5	5
Citalopram (20 mg)	1	2
Fluoxetin (20, 40 mg)	0	2
Paroxetin (20 mg)	1	0
Cipralex (60 mg)	1	0
Seralin (50 mg)	0	0
Atypical neuroleptic medication	3	3
Quetiapin (25, 50, 150, 300 mg)	1	2
Truxal (15 mg)	2	1

The study has been approved by the local ethics committee (Ethical approval number: 2014-374). We assumed medium to large aerobic exercise-induced effect sizes for the main primary outcome depression severity. Thus, a sample size of 35 patients has been estimated. Thereby, a significant effect (*p* < 0.05) can be detected with a statistical power of 90%. Considering an expected dropout rate of approximately 20%, at least 42 patients had to be recruited (5). All subjects signed an informed written consent after receiving all relevant study-related information.

### Inclusion and Exclusion Criteria

Inclusion criteria for the patients was a clinical diagnosis of a single episode of one of the following mood affective disorders according to the International Statistical Classification of Diseases and Related Health Problems 10th Revision (ICD-10) (21) criteria:

F32.1: major depressive disorder, single episode, moderate
F32.2: major depressive disorder, single episode, severe without psychotic features
F33.1: major depressive disorder, recurrent, moderate
F33.2: major depressive disorder, recurrent severe without psychotic features

Exclusion criteria were any further psychiatric diagnoses such as (a) recurring depression, (b) eating disorders such as anorexia, bulimia, or binge-eating, (c) addictions (alcohol) or current detoxification treatment, (d) schizophrenia, (e) bipolar disorder, and (f) panic disorders with or without agoraphobia or somatic disorders including (a) CVDs, (b) stroke or thrombosis, (c) epilepsia or other neurological disorders, (d) pulmonary diseases, (e) diabetes, or (f) obesity (BMI ≥ 30).

### Testing Procedures

#### Assessment of Depression Severity

For the determination of the individual’s depression severity, the Beck-Depression-Inventory-II (BDI-II) was applied. This questionnaire yields reliable, internally consistent and valid scores to detect depression (22) and describes the somatic and cognitive-emotional symptoms of the disease (23). The inventory can be subdivided into two parts: the negative view of self and the somatic/physical function. The first subdivision comprises six items related to sense of failure, guilt, punishment, self-dislike, self-accusation, and body image changes while the latter reflects the cognitive-emotional and somatic aspects of depression such as social withdrawal, work difficulty, insomnia, fatigability, loss of appetite, somatic preoccupation, and loss of libido (23). It is scored by summoning the highest ratings for each of the 21 symptoms. Each symptom is rated on a 4-point scale ranging from 0 to 3, total scores can range from 0 to 63 (24). Depending on the sum of the questions, the severity of the disorder can be assessed. According to Knaster et al., the following scores represent the severity index of depression (23): 0–9: no depression; 10–18: mild depression; 19–29: moderate depression; and 30–63: severe depression.

#### Arterial Stiffness

Markers of central hemodynamics (central systolic and diastolic blood pressure, pulse pressure), pulse wave reflection (AIX@75), and pulse wave velocity (PWV), a more direct marker of arterial stiffness, were obtained using an oscillometric Mobil-O-Graph^®^ PWA Monitor device (I.E.M GmbH, Germany) with integrated ARCSolver^®^ software. The measurements of arterial stiffness and central hemodynamics using the oscillometric method stand in good agreement with the conventional tonometric method (25) and have been invasively validated (26). The blood pressure cuff was placed on the left upper arm while the patient was lying in a resting position. The first of three measurements was performed after 5 min at resting period. From the measurements, central blood pressures, crude Aix, and AIx@75 as well as PWV were extracted. The two following measurements were performed at 2-min intervals. After data readout, every measurement was reviewed for erroneous values. The mean of all three measurements was taken, unless PWV differed >0.5 m/s. In this case, the two values closest to each other were selected.

#### Maximal Exercise Testing

Exercise tests were conducted on a bicycle ergometer (ErgoSelect 300, Ergoline) to determine maximal heart rate (HRmax) and maximal oxygen uptake (VO_2max_). The patients underwent a ramp-protocol with a regular increase of intensity of 10 W/min, starting at 25 W. During exercise testing, breath-by-breath spirometric gas-exchange data (Metamax 3b, Cortex, Leipzig, Germany), heart rate (HR) (Polar Electro Oy, Kempele, Finland), and ratings of perceived exertion were collected (27).

#### Exercise Intervention

Both HILV and MCT groups trained three times a week for a period of 4 weeks. In total, 12 training sessions were completed, whereby a minimum of 11 sessions were required for inclusion in the analysis. HILV absolved a Wingate-based interval protocol of 25 repetitions of 30-s high-intensity intervals at 80%VO_2max_ followed by 30 s of complete rest (remaining seated on the bicycle) (28). Including the warm-up and cool-down period of 5 min each, one session lasted 35 min. MCT cycled for 20 min at a constant pace of 60% of their individual VO_2max_. Their training session included the same warm-up and cool-down as the HILV group. For allowing the comparison between groups, the two training protocols were designed to be calorically equivalent.

### Statistics

Indices of central hemodynamics, arterial stiffness, and BDI-II score are given as means with SDs. Analyses of covariance (ANCONVA) were computed to adjust between-group effects for potential baseline differences (29). To estimate practical relevance of the ANCOVA between-group effects, effect sizes (partial eta squared, $\eta_{p}^{2}$) were additionally calculated. According to Cohen et al. (30), an $\eta_{p}^{2}\geq 0.01$ indicates a small, ≥0.06 a medium, and ≥0.14 a large effect. Standardized mean differences [Cohen’s *d*, trivial: SMD < 0.2, small: 0.2 ≤ SMD < 0.5, moderate: 0.5 ≤ SMD < 0.8, large SMD ≥ 0.8 (31)] were calculated for each group and variable.

The absolute and percentage differences as well as the standardized mean differences (Cohen’s *d*) in the change scores between HILV and MCT from pre- to post-testing were also calculated together with 90% confidence intervals according to the magnitude-based inference approach (32). These calculations were adjusted for pre-test values. A practically worthwhile change was assumed when the difference score was at least 0.2 of the between-subject SD (33). The probability for an effect being practically worthwhile was calculated according to the magnitude-based inference approach using the following scale: 25–75%, possibly; 75–95%, likely; 95–99.5%, very likely; >99.5%, most likely (32). The default probabilities for declaring an effect practically beneficial were <0.5% (most unlikely) for harm and >25% (possibly) for benefit (33). All calculations were conducted using a published spreadsheet in Microsoft^®^ excel (34).

## Results

### Depression Severity Index

In the ANCOVA analysis, taking baseline values into account, we found relevant but small interaction effects on depression severity reduction (*p* = 0.07; $\eta_{p}^{2}=0.10$). Pairwise comparison of BDI-II showed large effects in both groups [HILV: pre: 28.8 (9.5), post: 15.5 (8.5), SMD = 1.48; MCT: pre: 33.8 (8.5), post: 22.6 (7.5), SMD = 1.40] (Table 3). HILV showed a likelihood of meaningful effect of 85% likely beneficial compared to MCT (Table 4). In relation to the BDI-II, the reduction for HILV corresponds to a change from moderate to mild depression, while MCT went from severe to moderate.

**Table 3:** Pre and post intervention results of both groups for peripheral and central vessel parameters and arterial stiffness parameters.

		Pre mean (SD)	Post mean (SD)	SMD Cohen’s d	ANCOVA	
					*p*	$\eta_{p}^{2}$
BDI-II	HILV	28.8 (9.5)	15.5 (8.5)	1.48	0.07	0.10
	MCT	33.8 (8.5)	22.6 (7.5)	1.40		
pSBP [mmHg]	HILV	120.1 (10.5)	119.4 (12.6)	−1.50	0.94	0.0
	MCT	115.9 (11.2)	116.0 (12.3)	−0.01		
pDBP [mmHg]	HILV	78.1 (8.0)	76.5 (8.8)	0.19	0.59	0.01
	MCT	73.4 (10.4)	72.9 (13.2)	0.04		
cSBP [mmHg]	HILV	112.7 (10.8)	111.3 (13.2)	0.12	1.0	0
	MCT	109.4 (12.0)	108.9 (13.5)	0.04		
cDBP [mmHg]	HILV	78.7 (8.3)	77.0 (8.9)	0.20	0.42	0.21
	MCT	74.0 (10.3)	74.0 (13.2)	0		
cPP [mmHg]	HILV	34.0 (6.2)	34.3 (8.7)	−0.04	0.94	0
	MCT	35.5 (9.1)	35.0 (8.3)	0.06		
AIx [%]	HILV	25.9 (12.4)	24.5 (12.6)	0.11	0.39	0.02
	MCT	34.1 (13.8)	23.0 (15.0)	0.77		
AIx@75 [%]	HILV	19.5 (10.9)	18.6 (10.5)	0.08	0.17	0.06
	MCT	23.0 (13.6)	14.6 (13.8)	0.61		
PWV [m/s]	HILV	6.1 (1.1)	6.1 (1.2)	0	0.57	0.01
	MCT	5.9 (0.9)	5.9 (1.0)	0		
VO_2max_ [ml/min/kgBW]	HILV	34.7 (8.5)	35.6 (8.7)	−0.10	0.63	0.08
	MCT	30.1 (6.0)	30.7 (6.3)	−0.10		

*All results depicted as mean and SD*.

*BDI-II, Beck Depression Inventory-II; pSBP, peripheral systolic blood pressure; pDBP, peripheral diastolic blood pressure; cSBP, central systolic blood pressure; cDBP, central diastolic blood pressure; cPP, central pulse pressure; AIx, Augmentation index; AIx@75, Augmentations index corrected for 75 beats per minute; PWV, pulse wave velocity; VO_2max_, maximum oxygen uptake; HILV, high-intensity low volume*.

**Table 4:** Parallel group trials for HILV and MCT.

Maximal parameters	Differences in means	Standardized mean difference [90% CI]	Probability for a practically worthwhile effect
BDI-II Score			
HILV vs. MCT	−4.3 [−8.3; −0.4]	−0.45 [−0.85; −0.04]	85%; likely beneficial
pSBP [mmHg]			
HILV vs. MCT	0.4 [−5.7; 6.5]	0.03 [−0.50; 0.57]	24%; unlikely beneficial
pDBP [mmHg]			
HILV vs. MCT	−1.2 [−5.0; 2.7]	−0.12 [−0.51; 0.27]	38%; possibly beneficial
cSBP [mmHg]			
HILV vs. MCT	0.0 [−6.5; 6.5]	0.00 [−0.55; 0.55]	28%; possibly beneficial
cDBP [mmHg]			
HILV vs. MCT	−1.7 [−5.5; 2.0]	−0.18 [−0.56; 0.21]	48%; possiblybeneficial
cPP [mmHg]			
HILV vs. MCT	0.2 [−4.0; 4.5]	0.03 [−0.51; 0.57]	25%; unlikely beneficial
AIx [%]			
HILV vs. MCT	4.1 [−0.5; 12.7]	0.29 [−0.32; 0.90]	9%; unlikely beneficial
MCT vs. HILV	−4.1 [−12.7; 4.5]	−0.29 [−0.90; 0.32]	61%; possibly beneficial
AIx@75 [%]			
HILV vs. MCT	5.5 [−1.4; 12.3]	0.43 [−0.11; 0.97]	3%; very unlikely beneficial
MCT vs. HILV	−0.8 [−9.1; 7.4]	−0.06 [−0.71; 0.59]	37%; possiblybeneficial
PWV [m/s]			
HILV vs. MCT	−0.1 [−0.3; 0.2]	−0.07 [−0.30; 0.16]	18%; unlikely beneficial
VO_2max_ [ml/min/kgBW]			
HILV vs. MCT	0.6 [−1.2; 2.5]	0.08 [−0.15; 0.31]	20%; unlikely beneficial

*Parallel group trials difference in means as well as standardized mean differences (90% confidence intervals) is given for BDI-II scores, central hemodynamic as well as arterial stiffness parameters. The probability for an effect being practically beneficial was calculated according to the magnitude-based inference method*.

*BDI-II, Beck Depression Inventory-II; pSBP, peripheral systolic blood pressure; pDBP, peripheral diastolic blood pressure; cSBP, central systolic blood pressure; cDBP, central diastolic blood pressure; cPP, central pulse pressure; AIx, Augmentation index; AIx@75, Augmentations index corrected for 75 beats per minute; PWV, pulse wave velocity; VO_2max_, maximum oxygen uptake; HILV, high-intensity low volume*.

### Arterial Stiffness

Taking baseline values into account, pairwise comparison of peripheral (p) SBP revealed large interaction effects in HILV [pre: 120.1 (10.5), post: 119.4 (12.6), SMD = −1.5]. MCT showed moderate effects in pairwise comparison for AIx [pre: 34.1 (13.8), post: 23.0 (15.0), SMD = 0.77] and AIx@75 [pre: 23.0 (13.6), post: 14.6 (13.8), SMD = 0.61] (Table 3). The likelihood of meaningful effect was 61% possibly beneficial and 37% possibly beneficial, respectively, compared to HILV (Table 4).

### Physical Fitness

ANCOVA found moderate interaction effects on VO_2max_ (*p* = 0.63; $\eta_{p}^{2}=0.08$). Pairwise comparison showed trivial effects in both groups (SMD = −0.10). HILV showed an unlikely beneficial effect over MCT (20%).

## Discussion

The aim of our study was to compare the effects of a 4-week exercise intervention of continuous and discontinuous aerobic exercise at different intensities on depression severity as well as arterial stiffness and central hemodynamics in unipolar depression. Our results confirm previous findings which describe positive effects of aerobic exercise as adjuvant treatment for patients with clinical depression (35, 36). We are able to show, for the first time, that the impact on depression severity and vascular health depend on exercise intensity and modality. Both exercise interventions lowered depression scores whereby HILV was notably more effective than continuous exercise performed at moderate intensity (MCT). On the other hand, MCT had a greater effect on reducing peripheral arterial stiffness than HILV.

The pathophysiology of depression and CVD show several similarities including metabolic and immunomodulatory dysregulation, autonomic dysfunction, and sympathetic overstimulation (37) as well as increased arterial stiffness (38). Arterial stiffness causes an increase in blood pressure, which leads to an additional burden for the vascular system and may, in turn, induce further stiffening of the arteries (39). The stiffened arteries foster excessive pressure and pulsatile flow in the peripheral vascular system, inducing vascular remodeling and the development of atherosclerosis (12). Hence, arterial stiffness may in part explain the relationship between vascular dysfunction and depression, especially since several biological abnormalities have been found in the pathophysiology of depression including dysregulation of serotonin transmission, increased pro-inflammatory cytokines, and decreased neurogenesis (16). As many studies have proven, regular exercise has the potential to counteract vascular disease by improving endothelial function, containing inflammatory processes, and reducing sympathetic overdrive (40–43). Regular exercise reduces pressure wave reflection and improves vascular function by increasing blood flow and shear stress of the vascular wall leading to increased nitric oxide availability and peripheral arterial dilatation. The extent of these exercise-induced adaptations seems intensity-dependent (28). In our study, both exercise programs improved peripheral vascular function by decreasing AIx and, in turn, lowering cardiovascular risk. The effect in MCT was notably superior to HILV. Maximal fitness parameter VO_2max_ showed same effect sizes in both groups with no advantage for either exercise intervention. These findings seem contrary to findings comparing the effects of high-intensity interval training (HIT) protocols to MCT in healthy subjects (19). A previous review concluded that HIT is superior in improving endothelial function and resting blood pressure compared to MCT (28). Improvements in physical fitness have been shown to be dependent of the applied exercise protocol (44), with the most beneficial physiological adaptations induced by low volume interval programs of long duration (45). An additional important determinant of interval efficacy is the work-to-rest ratio, in which the interventions with longer and adequate rest periods between the bouts of high intensity show greatest improvements in cardiovascular and functional fitness as well as health-related quality of life and physical functioning (45). Based on these findings, one may speculate why HILV was not more effective in our study setting. The stimulus from HILV may not have been high or long enough to trigger adaptations of vascular integrity and an increased VO_2max_. Another kind of aerobic interval training protocol with longer bouts of high intensity and longer resting periods might be more effective in creating the necessary endothelial stimulation to reduce peripheral arterial stiffness. The overall duration of 4 weeks may have been too short for more pronounced effects on vascular health. It may also be debated that in a stressful state of constant sympathetic overdrive such as depression, additional high-intensity interval stimulation of the sympathetic nervous system by HILV does not translate into optimal improvement of vascular function and integrity.

As mentioned, a clear link has been made between cardiovascular risk and depression (46) and etiological studies suggest that the presence of depression doubles the risk of developing new-onset CVD (47). Since AIx is an established, independent cardiovascular risk predictor (13), and both our intervention groups showed lower post-exercise AIx values, we can therefore presume that the risk of experiencing a cardiovascular event was lowered in the participants, whereby MCT was more effective than HILV.

Even though MCT was more effective in improving AIx, both exercise programs showed large effects on lowering depression scores, with HILV being notably superior to MCT. On a molecular level, these findings may be explained by an exercise-induced release of brain-derived neurotrophic factor (BDNF), a neurotrophin which has been negatively correlated to depression-related personality traits in healthy subjects (48). The fundamental involvement of BDNF in psychiatric disorders has been confirmed by a meta-analysis which correlated decreased serum BDNF to major depressive disorders (49). Physical exercise has been shown to be able to increase low serum levels in depressed patients to normal levels (6), whereby the increase of BDNF was associated to the intensity of the applied exercise (50). Higher exercise intensities are able to increase circulating BDNF more than moderate intensities (50). This intensity-dependent mechanism may be one possible explanation as to why HILV was slightly more effective in lowering BDI-II scores than MCT.

Our results indicate that regular aerobic exercise should be included as a non-pharmaceutical treatment option in unipolar depression. Studies have shown that antidepressants are efficient in acute depression (51, 52), adherence levels nonetheless remain low (53) and about 30% of patients do not achieve remission after four different antidepressant treatment trials (54). These outcomes are mainly due to patients’ concerns about side effects and potential addiction (53). Furthermore, surveys consistently demonstrate that patients prefer non-pharmacological therapies to medication (55, 56). This calls for innovative non-pharmaceutical treatment options which do not possess the potential side effects of regular and long-term use of medication. Our results suggest that regular aerobic exercise should be prescribed and performed under professional supervision as preventive as well as rehabilitating treatment options. Additionally, our results confirm that regular exercise has beneficial effects on arterial stiffness, indicating the potential of regular exercise to reduce cardiovascular risk in unipolar depression. Our findings have a high clinical relevance and should be considered during clinical practice and in treatment prescription.

Some limitations of the study need to be addressed. Depression is a complex disease, and treatment strategies must be individualized to achieve optimal results. Since all participants were inpatients at the same clinic, we were able to perform our measurements in a closed setting, thus minimalizing any exterior factors which might have influenced the results. This closed setting did not allow for the assessors nor the mode of exercise to be blinded to the intervention. The motivation to take part in the additional exercise treatment was relatively low. 99 (out of 170) patients did not want to participate in the study. This number is explained by the fact that they were hospitalized for unipolar depression and lack of motivation is part of the disease entity. During their stay at the clinic, all inpatients received similar additional non-exercise therapy provided by the clinic. Changes of medication may have direct effects on the outcome. However, MCT was used as a control group with comparable changes in medication. Intervention duration was limited by the duration of stay at the clinic. Additionally, despite group allocation based on anthropometric parameters and BDI-II scores, notable baseline differences in AIx need to be mentioned. The variability of AIx spot measurements is known to be relatively high despite taking the mean of three measurements. Baseline values were applied as covariates, adjusting results for potential baseline differences. The sample size of the study might be considered low. In our group analysis, we refrained from interpreting our data on the basis of mere conventional *p*-values to estimate relevant between-group effects, as *p*-values do not sufficiently allow for continuous estimation of relevant interventional effect sizes (20). These would have failed to reach significance, possibly affected by the small sample size of this study.

## Conclusion

Our results in inpatients with unipolar depression demonstrate the beneficial effects of short-term exercise regimes on reduction of depressive symptoms and cardiovascular risk during the hospital stay. Both training regimes showed large effects on the reduction of depressive symptoms. While HILV was more effective in lowering depression severity, MCT was more effective in additionally lowering peripheral arterial stiffness. Future studies have to investigate whether a combination of both our exercise modalities can optimize therapeutic targets for disease severity and CVD reduction. Exercise should be considered an important strategy for preventive as well as rehabilitative treatment in depression.

As a future perspective, patients will have to continue with their additional exercise therapy after discharge and need infrastructure to maintain adherence. More research is warranted to investigate feasibility of regular exercise training in a home-based setting under no or at least less supervision. Most importantly, a high degree of motivation and adherence to exercise therapy needs to be established. The use of e-health platforms, mobile App support, and/or exergaming are promising tools to increase motivation and adherence to regular exercise and physical activity in patients with recurrent depression.

It remains to be elucidated how exercise programs can be routinely implemented into hospital and home-based care and how this can be financed by the health care system. Moreover, prospective studies are warranted to define the effect of different exercise modes on long-term disease progression and cardiovascular outcome.

## Ethics Statement

The study has been approved by the local ethics committee (Ethical approval number: 214-374). All subjects signed an informed written consent after receiving all relevant study-related information.

## Author Contributions

Study design: HH and LD; data collection: AM; statistical analysis: AM and LD; manuscript preparation: HH, AM, and LD; manuscript revision: OF, AS-T, LZ, and JB.

## Conflict of Interest Statement

All authors do not have any conflict of interest concerning the present study including data acquisition, manuscript writing, and data presentation.
